# An Empirical Investigation into the Impact of Social Media Fitness Videos on Users’ Exercise Intentions

**DOI:** 10.3390/bs14030157

**Published:** 2024-02-21

**Authors:** He Yin, Xin Huang, Guangming Zhou

**Affiliations:** School of Journalism and Communication, Wuhan University, Wuhan 430072, China; yinhe_yh@whu.edu.cn (H.Y.); huang-xin@whu.edu.cn (X.H.)

**Keywords:** social media fitness video, social media fitness influencer, exercise intentions, stimulus–organism–response (S-O-R) model, source credibility theory

## Abstract

Social media fitness influencers are driving the maturation of online fitness, which is especially significant in the current era of globally decreasing levels of physical activity. However, there is a paucity of research on online fitness videos, and the mechanism of influence of fitness videos on exercise intention is not well understood. Therefore, based on the stimulus–organism–response (S-O-R) theoretical framework, this study extends the source credibility theory to the field of fitness and adds an examination of the content quality and electronic word-of-mouth of fitness videos to explore how fitness videos motivate users to participate in physical exercise. Through an online survey, 367 valid samples were collected and validated using a structural equation model. The results showed that the three elements contained in source credibility theory have inconsistent importance in the fitness field, with trustworthiness being the most important, followed by attractiveness, and the influence of expertise is not significant. In summary, the attributes of social media fitness influencers, including trustworthiness and physical attractiveness, as well as the content quality and electronic word-of-mouth of their fitness videos, may lead to viewers’ trust and perception of the usefulness of the videos and, furthermore, lead to exercise intention.

## 1. Introduction

Active exercise can help people to prevent and manage non-communicable diseases, with significant benefits for the heart, body, and mind. However, lack of exercise has a negative impact on people’s physical health, community wellbeing, and quality of life, on a global scale [1]. Therefore, in addition to calling for more exercise, it is equally important to find ways to motivate exercise and maintain it [2]. Today, internet technology is more mature and can provide people with the latest and useful information. Social media, in particular, has become a common means for people to seek and communicate health information [3]. At the same time, previous research has found that social media may induce changes in exercise behavior through the content posted [4,5,6].

“The internet is ushering in an era where the role of personal influence will attain unprecedented prominence” [7]. The internet and social media platforms give every user an equal opportunity to express and share, but they also lead to information overload and an uneven quality of information. As a result, social media influencers are increasingly being relied upon. They are often highly followed, have expertise in a specific field, and have the ability to create valuable content and provide useful advice. Thus, similar to traditional media figures, social media influencers are also held up as models of observation and learning who can influence their audience’s intentions and beliefs [8]. In the field of health, a combination of health communicator and influencer strategies are more suitable for the media consumption habits in the mobile internet era, and social media fitness influencers also participate in the construction of health communication patterns in the digital era, as health communicators [2,9]. The phenomenon of following an influencer to exercise is prevalent in today’s social media context, where followers have access to a wide selection of free fitness videos, especially since the COVID-19 pandemic has changed people’s lifestyles. At present, there is not much research on fitness videos and social media fitness influencers.

In fitness-related research, there has been a growing interest in fitness applications and related health behaviors in recent years [10,11], with the focus primarily on the performance expectations, technical characteristics, and user stickiness of applications; however, limited attention has been given to exploring the influence of social media fitness influencers and social media fitness videos on exercise intentions. In the existing studies, the stimulus–organism–response (S-O-R) theoretical framework (this theoretical framework assumes that aspects of the environment work together as a stimulus to influence people’s internal states, thereby inducing individual perceptions that then influence their psychological and behavioral responses) has often been applied to research in the fields of commercial marketing and tourism to explore how stimulus factors affect consumer behavior through the actions of organisms. Few studies have used the S-O-R framework as a basis for health- and fitness-related research. In fact, this framework is also suitable for research in the field of online fitness. Some elements in fitness videos can be regarded as stimulating factors to motivate viewers to exercise. According to the source credibility theory, the trustworthiness (TRU), expertise (EXP), and attractiveness (ATT) of the spokesperson are very important to the persuasiveness of the message [12], and a large number of studies have applied the source credibility structure to the study of social media influencers, confirming the positive effects of these three factors on perceived trust and usefulness [13,14,15]. However, does this influence mechanism also exist in the field of fitness? There is not enough research on this question. In addition to the trainers in fitness videos, the quality of the information and content may also change people’s attitudes and beliefs [16]. Moreover, reviewing previous studies on fitness videos, we found that these studies have neglected the impact of electronic word-of-mouth (eWOM), which is closely related to consumer behavior in marketing practices and has a strong impact on consumers’ purchasing decisions [17]. Hence, one of our main research objectives was to determine whether eWOM also has a significant impact in the field of fitness media information consumption behavior. Perceived trust and perceived usefulness are important determinants of behavioral intention [18,19]. When users trust a fitness video and think that it is useful for their body shape, they may consider using it for exercise. Therefore, this study considered these two factors as organism factors and examined whether they play a mediating effect between fitness videos and exercise intention.

In general, in order to further understand the relationship between fitness videos and users’ exercise intentions, this study empirically investigated which factors in fitness videos drive users’ cognitive changes that lead to their perception of trust and usefulness of the information and, subsequently, to their intention to exercise. Specifically, we constructed a relationship model of social media fitness influencer attributes, content quality (CQ), electronic word-of-mouth (eWOM), information credibility (INC), perceived usefulness (PU), and exercise intention (EXI), based on the S-O-R theoretical framework, to explore the effects of online fitness videos on users’ exercise intentions.

## 2. Theoretical Background

### 2.1. The S-O-R Model

The stimulus–organism–response model (S-O-R model), proposed by Mehrabian and Russell [20] in 1974, posits that aspects of the environment work together as a stimulus (S) to influence people’s internal states (O), thereby inducing individual perceptions that then influence their psychological and behavioral responses (R) [21]. The S-O-R model has been widely used in the field of consumer behavior research. For example, a study on the impact of live broadcast marketing on consumers’ purchase intentions considered live streaming features as a stimulating factor that could positively affect consumers’ perceived value and trust and indirectly facilitate consumers’ purchase intentions [22]. Kim and Johnson [23] used the S-O-R model as a theoretical framework to investigate the impact of brand-related UGC on consumer behavior, and they proposed that brand-related UGC as a stimulus would cause emotional and cognitive responses in consumers and then drive a series of behavioral responses, such as information pass-along, impulse buying, future purchase intention, and brand engagement. To clarify how social media users respond to fitness videos, the stimulus–organism–response model (S-O-R model) paradigm was used as the basis for this research model. We regarded the social media fitness influencer attributes, electronic word-of-mouth (eWOM), and content quality in fitness videos as stimuli that affect viewers’ perceptions, took the perceived credibility and usefulness of the information as organic factors, and used exercise intention to measure viewers’ behavioral responses.

### 2.2. Source Credibility Theory

Source credibility is defined as the extent to which a recipient of information believes that a source is trustworthy, competent, and reliable [24]. Ohanian [12] proposed that source credibility implies positive characteristics of the communicator, which will affect the acceptability of the information to the recipient. Hovland and colleagues [25] constructed the source credibility model and pointed out that two key determinants of source credibility are perceived expertise and trustworthiness. Source credibility theory is widely used in marketing research and is often used to examine the effectiveness of celebrity endorsements [26]. Ohanian [12] constructed scales of expertise, trustworthiness, and physical attractiveness in his research on celebrity endorsements, which established a scientific and effective way to apply this theory. Smith [27] applied source credibility theory to investigate celebrity endorsements in political elections and found that the trustworthiness, expertise, and attractiveness of endorsers were positively correlated with the image of their political parties. Djafarova and Trofimenko [28] focused on the credibility of micro-celebrity endorsements and proposed new dimensions of source credibility applicable to the online context, based on the existing source credibility model’s dimensions. Based on these, we took “expertise, trustworthiness and physical attractiveness” as the dimensions of social media fitness influencer attributes to be examined in this study.

## 3. Hypothesis Development

### 3.1. Social Media Fitness Influencers’ Attributes, Information Credibility, and Perceived Usefulness

#### 3.1.1. Social Media Fitness Influencers’ Attributes and Information Credibility

Information credibility is defined as the extent to which information is perceived to be believable [29]. This study considers information credibility as the credibility of the information provided in videos posted by social media fitness influencers.

Erdogan [30], in the study “Celebrity endorsement: a literature review”, described trustworthiness as the honesty, integrity, and believability of an endorser. Previous studies have shown that, on social media platforms, influencers’ trustworthiness is one of the most critical influencing factors on users’ attitudes and behavior [31,32]. Source credibility significantly affects consumers’ perceptions of the credibility of information [13,33,34].

Hovland and his associates [25] defined expertise as “the extent to which a communicator is perceived to be a source of valid assertions”; Ohanian [12] added that expertise is also referred to as “authoritativeness”, “competence”, “expertness”, or “qualification”. Crisci and Kassinove [35] found that having a more credible title, such as “Dr.”, will strengthen the impact of the communicator and their message. A previous study found that when a product has a perceived risk, consumers are more likely to be influenced by information from influencers who are market experts before making a purchase decision [36].

Patzer [37] defined physical attractiveness as the degree to which a communicator’s face and body image are pleasing to observe, and experimentally demonstrated that communicators with higher levels of physical attractiveness were more persuasive and perceived with more trust and as having greater expertise than communicators with lower levels of attractiveness. Information recipients develop positive beliefs toward attractive communicators, and attractiveness will enhance the effectiveness of persuasive information [38,39]. Physical attractiveness has been shown to be positively correlated with information credibility [13,40].

Based on previous studies, we established the following hypotheses to verify the influence of fitness influencers’ attributes on information credibility:

**H1a.**  *The trustworthiness of social media fitness influencers is positively correlated with information credibility*.

**H2a.**  *The expertise of social media fitness influencers is positively correlated with information credibility*.

**H3a.**  *The physical attractiveness of social media fitness influencers is positively correlated with information credibility*.

#### 3.1.2. Social Media Fitness Influencers’ Attributes and Perceived Usefulness

Perceived usefulness refers to the degree to which prospective users expect to increase their performance using a particular system, and it strongly influences people’s intentions and is a major factor in determining whether people use a particular system [19]. Perceived usefulness in this study refers to the extent to which users perceive the content of a fitness video on social media to be useful for their exercise.

Trust in the source of information will affect the recipient’s assessment of the usefulness of the information. A study on how travel vloggers’ YouTube videos influence the future behavior of tourists showed that source credibility has a significant impact on the perceived usefulness of information [15]. From the perspective of trust transfer, Hu et al. [41] showed that if social media users have enough trust in influencers, they will consider applications approved by those influencers to be highly useful.

Expertise consists of accumulated knowledge, skills, and competencies, and experts often have the ability to provide reliable advice that reduces costs, such as time and effort spent searching for information and evaluating products [42]. Tien et al. [43] found that source expertise greatly predicted cosmetic users’ evaluation of the usefulness of eWOM on social media, in that information providers with a high level of expertise were more able to convince other consumers.

Physically attractive people proved to be more likely to be effective communicators and, thus, more persuasive than less physically attractive people [44]. For social media users, the best proof of the usefulness of fitness videos’ content is the visual appearance of the social media fitness influencer, such as their body shape, skin, and energy. A physically attractive influencer will inspire the viewer’s desire to “want to be like him or her”, thus generating the willingness to exercise, as Durau et al. [2] defined the concept of “the motivating power”.

Based on previous studies, we established the following hypotheses to verify the influence of fitness influencers’ attributes on the perceived usefulness of their videos’ content:

**H1b.**  *The trustworthiness of social media fitness influencers is positively correlated with perceived usefulness*.

**H2b.**  *The expertise of social media fitness influencers is positively correlated with perceived usefulness*.

**H3b.**  *The physical attractiveness of social media fitness influencers is positively correlated with perceived usefulness*.

### 3.2. Content Quality of Fitness Videos, Information Credibility, and Perceived Usefulness

Content quality includes the accuracy, completeness, relevance, and timeliness of information [45]. High-quality information can help consumers to reduce risk and make better decisions, and consumers are more likely to have confidence that the information and the information provider are reliable [46]. Vila and Kuster [47] demonstrated that the information quality of a website can enable consumers to build more perceived trust. Saima and Khan [48] proposed that, in social media marketing interactions, the quality of information provided by influencers significantly affects their credibility and consumers’ purchase intentions.

Lin and Lu [49] explored the factors that affect users’ acceptance or rejection of websites, and their results showed that websites that provide higher-quality information will be perceived as more useful by the user. The consumer’s decision-making process is energy-consuming, and if a website does not provide clear, complete, and current information, consumers will abandon it [50]. Effective information will lead to the internalization of behavior, and the more specific the information, the easier it is to provoke changes in individual behavior [51].

Based on previous studies, we established the following hypotheses to verify the influence of the content quality of fitness videos on the credibility of the videos’ information and the perceived usefulness of their content:

**H4a.**  *The content quality of fitness videos is positively correlated with information credibility*.

**H4b.**  *The content quality of fitness videos is positively correlated with perceived usefulness*.

### 3.3. Electronic Word-of-Mouth of Fitness Videos, Information Credibility, and Perceived Usefulness

The increasing maturity of internet technology and social media has facilitated the development of online WOM, that is, electronic word-of-mouth (eWOM); eWOM is not limited by time and space, providing online consumers with a platform to connect the whole world via the internet, where they can obtain a large amount of information on product experiences from experienced consumers as a reference [52]. Furthermore, eWOM can provide consumers with high levels of transparency on market and product information, and it is easier to understand than seller information [53]; so, consumers search and read online recommendations in the pre-purchase phase, mainly to save time and make better decisions [54]. Experienced consumers are considered to have no vested interest in a product; their personal experiences and opinions create vicarious experiences for users, and thus, they are perceived as trustworthy [13,55].

eWOM is becoming the main reference information for people’s purchasing decisions [56]. Huang’s research showed that users tend to rely on social cues as a reference for perceptions of usefulness before experiencing an information system [57]. Positive word-of-mouth from users who have used it will convince prospective users that the system is a useful tool. In addition, people may try to observe others and follow them in making decisions in order to reduce uncertainty and avoid information asymmetry [58].

Based on previous studies, we established the following hypotheses to verify the influence of eWOM about fitness videos on the credibility of the videos’ information and the perceived usefulness of their content:

**H5a.**  *The eWOM of fitness videos is positively correlated with information credibility*.

**H5b.**  *The eWOM of fitness videos is positively correlated with perceived usefulness*.

### 3.4. Information Credibility and Exercise Intention

Behavioral intention is considered to be the most effective and closest predictor of actual behavior [59]. Many studies have confirmed the positive correlation between credibility and behavioral intention, especially in business transactions. For example, Choi et al. confirmed that believable information and services are factors in the intention to use mobile apps for travel-related purposes [60]. Research by Kim et al. [61] showed that trust has a strong positive impact on consumers’ online transactions. The degree of trust is an important variable in predicting behavioral intention, and the degree of trust that users have for websites is a key antecedent for their online purchases [62]. Based on these research foundations to predict the effect of credibility on behavioral intention, we established the following hypothesis:

**H6.**  *Information credibility is positively correlated with exercise intention*.

### 3.5. Perceived Usefulness and Exercise Intention

The influence of perceived usefulness on behavioral intention has been reported in various studies. Scholars found that information quality has an indirect effect on continuous use intention, mediated by perceived usefulness, in a study on the determinants of the continuous use intention of food-delivery applications [63]. In addition, a study on the acceptance and use of mobile learning services by Malaysian university students found that usage intention was facilitated by usefulness [64]. Chen et al. proposed that perceived usefulness can greatly predict the continuous usage intention of mobile service users [65]. Based on previous research on the relationship between perceived usefulness and behavioral intention, we established the following hypothesis:

**H7.**  *Perceived usefulness is positively correlated with exercise intention*.

**Figure 1:**
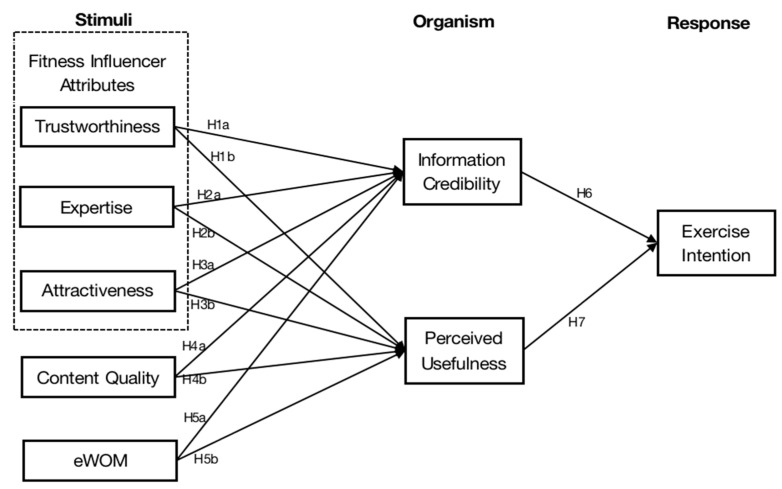
The research model.

Based on the discussion above, we proposed a conceptual model (see Figure 1).

## 4. Methodology

### 4.1. Measures

To test the research model and answer the research questions, we designed a questionnaire based on and adapted from previous studies. The first part of the questionnaire collected the demographic information of the participants, including gender, age, and level of education. The second part measured the variables in the study model using a 5-point Likert scale from 1 (“strongly disagree”) to 5 (“strongly agree”). Table A1 presents a detailed overview of the measurement scales, including all items.

Trustworthiness/expertise/attractiveness: The participants were asked to rate social media fitness influencers’ trustworthiness (Cronbach’s α = 0.960), expertise (Cronbach’s α = 0.943), and attractiveness (Cronbach’s α = 0.926), and we adapted Ohanian’s scale [12] for validating celebrity endorsers. Trustworthiness consisted of four items: trustworthy, sincere, honest, and dependable. Expertise consisted of four items: knowledgeable, experienced, skilled, and expert. Attractiveness consisted of four items: classy, beautiful, sexy, and attractive.

eWOM: To measure the impact of eWOM, we adapted Abubakar and Ilkan’s scale [66], and participants were asked to indicate the extent to which eWOM affected their perceived trust and the perceived usefulness of social media fitness videos; one example item was “I often gather information from other fitness enthusiasts’ online reviews before using fitness videos for workouts” (Cronbach’s α = 0.931).

Content quality: To measure the effect of the content quality of social media fitness videos on viewers, we adapted a scale developed by Magno [67], consisting of four items: reliable, updated, accurate, and high-quality. The participants were asked to evaluate the content quality of fitness videos (Cronbach’s α = 0.923).

Information credibility: We adapted a scale from Lederman et al. [68] and asked the participants to indicate what they thought of the information provided by the fitness videos: “I think fitness video information is truthful” (1 = strongly disagree; 5 = strongly agree) (Cronbach’s α = 0.962).

Perceived usefulness: Measures of perceived usefulness were adapted from Nagy [69] and Davis [70] and consisted of three items. The participants were asked to rate the effectiveness of the fitness videos; one example item was “Using fitness videos helps me improve my fitness efficiency” (Cronbach’s α = 0.950).

Exercise intention: The measure of exercise intention was adapted from Bhattacherjee and Sanford [24]; the participants were asked to indicate whether they intended to exercise after watching a fitness video. One example item was “I intend to use this fitness video to exercise in the near future” (Cronbach’s α = 0.881).

### 4.2. Data Collection and Sample

The questionnaire was distributed through Wenjuanxing (www.wjx.cn, accessed on 25 October 2023), a professional online questionnaire survey platform. We sent questionnaires via social media to potential participants from China. We set screening questions in the questionnaire to check whether they had watched fitness videos on social media and whether they had experience using fitness videos for exercise. Potential participants would have to answer “yes” to participate in the survey, and unqualified participants were not allowed further access. Finally, we collected a total of 367 valid questionnaires after excluding invalid answers. Table 1 shows the sociodemographic characteristics of the participants, who comprised 240 females (65.40%) and 127 males (34.60%). The largest proportions of participants were 19–35 years old (43.05%) and 36–59 years old (40.87%), while smaller numbers were 18 years and under or 60 years and over. Among all of the participants, 26.70% had a high school education or below, 23.98% had a college education, 38.42% had a bachelor’s degree, and 10.90% had a graduate degree.

## 5. Data Analysis and Results

The study applied the partial least squares structural equation modeling (PLS-SEM) statistical approach and analyzed the data using SmartPLS 3.2.9 software (SmartPLS GmbH, Oststeinbek, Germany). When applying structural equation modeling (SEM), there are two types of methods to estimate the relationships hypothesized in the model: covariance-based techniques (CB-SEM), and variance-based partial least squares (PLS-SEM) [71]. PLS-SEM operates much like a “multiple regression analysis” [72]. Unlike CB-SEM, which “estimates model parameters so that the discrepancy between the estimated and sample covariance matrices is minimized”, PLS-SEM “maximizes the explained variance of the endogenous latent variables by estimating partial model relationships in an iterative sequence of ordinary least squares (OLS) regressions” [71]. CB-SEM is suitable for testing a theory in the confinement of a concise theoretical model, while PLS is generally more conducive to smaller samples and more complex models. PLS emphasizes prediction and is preferred for research aimed at the prediction and explanation of target constructs [71,73]. The evaluation of the model followed the two-step method of measurement model evaluation and structural model evaluation.

### 5.1. Measurement Model Evaluation

To assess the validity and reliability of the instrument, we examined whether the instrument met the commonly suggested criteria for measurement model assessment: indicator reliability, construct reliability, convergent validity, and discriminant validity. Indicator reliability was measured by item loadings, and the recommended threshold is 0.7 [74]. As shown in Table 2, the standardized factor loading of each item in this study was higher than 0.7. Construct reliability was tested by composite reliability (CR) and Cronbach’s alpha (CA), and Table 1 shows that all constructs had CR and CA above the recommended threshold of 0.7 [75], thus confirming construct reliability. The convergent validity was assessed by the average variance extracted (AVE), and the AVE values for all constructs in Table 2 were higher than 0.5, which means that all constructs had satisfactory convergent effectiveness [74]. The discriminant validity, which reflects the degree of uniqueness of the latent variable, was assessed by the square root of the average variance extracted (AVE). According to Table 3, the square roots of the AVE for all potential variables were higher than their intercorrelations, which indicates that the scales had high discriminant validity [76]. Hence, this study’s constructs can be further used to test the structural model.

### 5.2. Structural Model Evaluation

**Figure 2:**
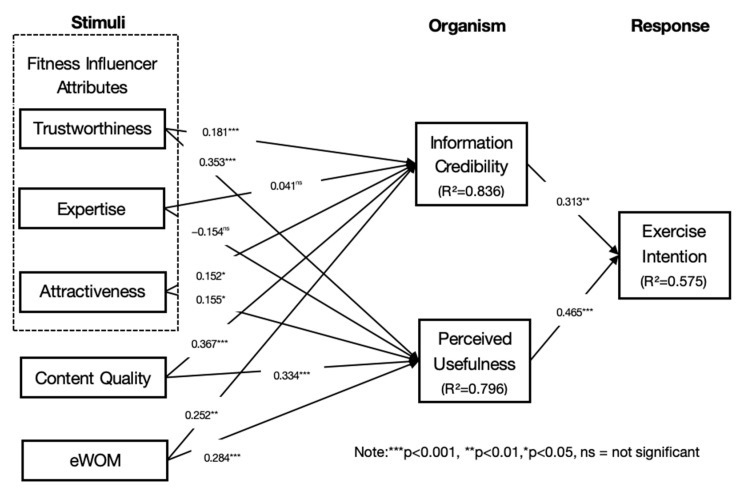
Results of the research model.

The most important criterion in the PLS path model for assessing the structural model is the coefficient of determination, or R^2^, of the endogenous latent variables; R^2^ is used to measure the model’s explanatory power [75]. As shown in Figure 2, the R^2^ value of information credibility is 0.836, the R^2^ value of perceived usefulness is 0.796, and the R² value of exercise intention is 0.575, indicating that the structural model provides adequate explanatory power.

We performed a bootstrapping procedure with 5000 resamples to estimate the statistical significance of path coefficients [74], with the results shown in Table 4.

Table 4 shows that all of the proposed hypotheses were accepted except for H2a and H2b. Specifically, trustworthiness was shown to have a positive effect on information credibility (β = 0.181, *p* < 0.001) and perceived usefulness (β = 0.353, *p* < 0.001), and H1a and H1b were supported. Attractiveness was shown to have a positive effect on information credibility (β = 0.152, *p* < 0.05) and perceived usefulness (β = 0.155, *p* < 0.05), with H3a and H3b supported. H4a and H4b proposed that content quality would be significantly associated with information credibility (β = 0.367, *p* < 0.001) and perceived usefulness (β = 0.334, *p* < 0.001), which was also accepted. eWOM was positively validated by information credibility (β = 0.252, *p* < 0.01) and perceived usefulness (β = 0.284, *p* < 0.001), so H5a and H5b were supported. Information credibility (β = 0.313, *p* < 0.01) and perceived usefulness (β = 0.465, *p* < 0.001) were shown to have a positive effect on exercise intention, supporting H6 and H7. However, the relationships between expertise and information credibility (β = 0.041, *p* = 0.513) and perceived usefulness (β = −0.154, *p* = 0.059) were found to be statistically insignificant, so H2a and H2b were not supported.

## 6. Discussion

According to the survey, the trustworthiness and physical attractiveness of social media fitness influencers have a positive impact on the perceived credibility and usefulness of their information; so, H1a, H1b, H3a, and H3b are all supported. However, the expertise of fitness coaches has no significant impact on the perceived credibility and usefulness of their information; so, H2a and H2b are rejected. On the one hand, this result suggests that social media fitness influencers’ trustworthiness and attractiveness may increase viewers’ trust in their videos’ information and improve their assessment of the usefulness of their fitness videos, which is consistent with previous findings [15,33,40,44]. In other words, the lessons and knowledge provided by a trustworthy, good-looking fitness influencer are more convincing. On the other hand, the fitness influencers’ expertise did not have a significant impact on users’ perceived trust and perceived usefulness of video information, which is inconsistent with previous findings [36,77]. A possible explanation for this is that viewers do not expect that the fitness trainer must be an authoritative expert. Instead, the trustworthiness of the trainer is most important—for example, whether they have the moral character of sincerity and honesty to provide true and accurate information, or whether they have enough professional ethics and, thus, give the audience reliable assistance in terms of fitness. The audience may believe that a sincere and trustworthy trainer is also trustworthy in their fitness classes. Findings from a study by McGinnies and Ward did show a supportive example of trustworthiness [78], which may support the findings of this study that trustworthy communicators are more persuasive, whether they are experts or not. In addition, viewers may find an intuitive fitness outcome more convincing than an authoritative title held by a trainer. A fitness trainer with an attractive physical appearance is more likely to have exceptional skills and extensive experience because the physical attraction is intuitive, allowing the viewer to see the outcomes of the exercise, and the trainer’s expertise can be tested through their exercise outcomes, online reviews of their fitness videos, or other more direct means than expertise. Moreover, there are many kinds of fitness videos on social media, and their quality is uneven. Most viewers lack professional training in fitness; so, it is difficult to judge whether the trainer is qualified and has enough professional knowledge just by watching online videos. Therefore, this cannot be the basis on which viewers judge whether the fitness course is effective or not.

The content quality of fitness videos has a significant impact on their information credibility and perceived usefulness, so H4 is supported. The results of this study show that the better the content quality, the higher the user’s trust assessment of the trustworthiness and usefulness of the fitness video. Specifically, clear, complete, and accurate high-value content can lead viewers to believe that using fitness videos can improve their awareness of fitness and their efficiency in reaching their fitness goals, thus increasing their intention to use fitness videos for exercise [47,50].

eWOM is also a predictor of information credibility and perceived usefulness. This proves that the opinions of other users will have a positive impact on an individual’s perception of fitness videos, and a positive recommendation from experienced users can increase individuals’ trust in fitness videos and make them more confident about the effects of their use, helping them to make the right decision. This finding is consistent with previous research [54,58], showing that when users do not have comprehensive information about fitness videos, they often follow others with more experience to help them make decisions, in the hope of achieving similar fitness outcomes.

Finally, as indicated in the findings, users’ intention to exercise is influenced by the credibility and perceived usefulness of the information; that is, the greater the user’s trust in and perception of the usefulness of the fitness video, the greater their intention to exercise, and behavioral intention is considered to be the most effective predictor of actual behavior [59,62,63].

## 7. Implications

### 7.1. Theoretical Implications

This study extends the application of the S-O-R model and source credibility theory to the field of online fitness, and it also enriches the research on social media influencers, further confirming that social media and social media fitness influencers may have a positive impact on users’ exercise intentions.

In previous research on online fitness, little attention has been paid to content quality and electronic word-of-mouth, yet the impact of these two factors on viewers’ attitudes and perceptions is significant. Thus, this study included an examination of the role of electronic word-of-mouth and content quality, clarifying the extent to which individual perceptions of fitness videos are influenced by the reviews of others and the quality of the videos’ information.

### 7.2. Practical Implications

Physical inactivity is a risk factor for premature mortality and several non-communicable diseases. This study found that fitness videos on social media may have a positive effect on individuals’ exercise intentions. Exercise with fitness videos is beneficial to adapt to diverse fitness needs in the digital age. It supports free exercise with fitness trainers anytime, anywhere, so as to help eliminate obstacles to exercise behavior, such as time and place restrictions, and the expense of working out, so as to motivate people to participate in and increase their exercise levels. This has important significance for the prevention of non-communicable diseases, the promotion of physical and mental health, and the development of healthy lifestyles.

## 8. Limitations and Future Directions

In addition to the important contributions of our research, there are some limitations. First of all, this article only explores the first time a viewer uses a fitness video for exercise, but continued use is also important, and one study has shown that continuity is more important than acceptance, because finding new customers can cost five times more than retaining existing customers [79]. Therefore, future studies could focus more on the process between first use and continued use. Secondly, this study focuses on the influence of fitness videos on exercise intention. Future studies could further expand the scope of investigation and add user factors, such as whether different group characteristics of users or their existing exercise habits will have an impact on their adoption and use of fitness videos. Third, our study used online questionnaires to collect information, and self-selection bias is likely to exist in such online surveys. Future studies could apply qualitative methods to supplement quantitative methods to support such studies.

## 9. Conclusions

This study empirically investigated the impact of fitness videos on social media platforms on users’ exercise intentions, and we constructed a model based on the S-O-R theoretical framework. Based on the application of source credibility theory, we found that social media fitness influencers’ trustworthiness and physical attractiveness, the fitness videos’ content quality, and electronic word-of-mouth all have positive effects on information credibility and perceived usefulness, and we also determined that information credibility and perceived usefulness play an important mediating role between fitness videos and exercise intention. That is, the combination of the trainer, the video’s content, and electronic word-of-mouth may lead to perceptions of the trustworthiness and utility value of the fitness video and, furthermore, to exercise intention. This result suggests that the factors influencing viewers’ intentions to exercise are not only the characteristics of the fitness videos themselves, but also information from external sources, namely, other people’s reviews. People usually look for social evidence to make sure their decisions are correct before trying something new [51], and the opinions of others often have an important influence on personal beliefs. In conclusion, this study expands the application field of the S-O-R model and source credibility theory, and it also expands the research on online fitness. It is of great significance for social media fitness influencers to understand their audience’s needs through online reviews and combine their own characteristics to optimize their fitness videos, so as to motivate more people to participate in physical exercise and develop a sustainable healthy lifestyle.

## Figures and Tables

**Table 1:** Sociodemographic characteristics of the participants (n = 367).

Item	Content	Frequency	Percentage
Gender	Male	127	34.60%
	Female	240	65.40%
Age	Aged 18 and under	27	7.36%
	19–35	158	43.05%
	36–59	150	40.87%
	Aged 60 and above	32	8.72%
Education	High school degree or below	98	26.70%
	College degree	88	23.98%
	Bachelor’s degree	141	38.42%
	Graduate degree	40	10.90%

**Table 2:** Factor loading, Cronbach’s α, composite reliability, and AVE.

Constructs	Item	Factor Loading	Cronbach’s α	Composite Reliability	AVE
Trustworthiness (TRU)	TRU1	0.952	0.960	0.971	0.894
	TRU2	0.945			
	TRU3	0.941			
	TRU4	0.944			
Expertise (EXP)	EXP1	0.891	0.943	0.959	0.854
	EXP2	0.949			
	EXP3	0.943			
	EXP4	0.911			
Attractiveness (ATT)	ATT1	0.937	0.926	0.947	0.819
	ATT2	0.916			
	ATT3	0.907			
	ATT4	0.857			
Content Quality (CQ)	CQ1	0.932	0.923	0.946	0.814
	CQ2	0.903			
	CQ3	0.854			
	CQ4	0.918			
Electronic Word-of-Mouth (eWOM)	eWOM1	0.862	0.931	0.948	0.784
	eWOM2	0.928			
	eWOM3	0.888			
	eWOM4	0.845			
	eWOM5	0.902			
Information Credibility (INC)	INC1	0.946	0.962	0.971	0.870
	INC2	0.913			
	INC3	0.925			
	INC4	0.930			
	INC5	0.948			
Perceived Usefulness (PU)	PU1	0.961	0.950	0.968	0.910
	PU2	0.970			
	PU3	0.930			
Exercise Intention (EXI)	EXI1	0.876	0.881	0.926	0.806
	EXI2	0.916			
	EXI3	0.902			

**Table 3:** Results of discriminant validity (Fornell–Larcker criterion).

	ATT	CQ	EXI	EXP	INC	PU	TRU	eWOM
ATT	0.905							
CQ	0.883	0.902						
EXI	0.702	0.731	0.898					
EXP	0.868	0.882	0.691	0.924				
INC	0.838	0.884	0.714	0.831	0.933			
PU	0.794	0.844	0.735	0.769	0.862	0.954		
TRU	0.745	0.806	0.617	0.799	0.795	0.807	0.945	
eWOM	0.758	0.794	0.674	0.747	0.813	0.791	0.680	0.885

**Table 4:** Structural path analysis results.

Hypothesis	Relationship	Path Coefficients	T Statistics	*p*-Values	Result
H1a	TRU → INC	0.181	4.048	0.000	Supported
H1b	TRU → PU	0.353	6.039	0.000	Supported
H2a	EXP → INC	0.041	0.654	0.513	Not supported
H2b	EXP → PU	−0.154	1.889	0.059	Not supported
H3a	ATT → INC	0.152	2.264	0.024	Supported
H3b	ATT → PU	0.155	2.075	0.038	Supported
H4a	CQ → INC	0.367	4.119	0.000	Supported
H4b	CQ → PU	0.334	3.578	0.000	Supported
H5a	eWOM → INC	0.252	2.989	0.003	Supported
H5b	eWOM → PU	0.284	4.041	0.000	Supported
H6	INC → EXI	0.313	3.209	0.001	Supported
H7	PU → EXI	0.465	4.766	0.000	Supported

## Data Availability

Data are available upon special request from the corresponding author.

## Appendix A

**Table A1:** Measurement items.

Construct	Measurement Items
Trustworthiness	The influencer is trustworthy.
	The influencer is sincere.
	The influencer is honest.
	The influencer is dependable.
Expertise	The influencer is knowledgeable.
	The influencer is experienced.
	The influencer is skilled.
	The influencer is expert.
Attractiveness	The influencer is classy.
	The influencer is beautiful.
	The influencer is sexy.
	The influencer is attractive.
Content Quality	The content of fitness video is reliable.
	The content of fitness video is updated.
	The content of fitness video is accurate.
	The content of fitness video is high-quality.
eWOM	I often gather information from other fitness enthusiasts’ online reviews before using fitness videos for workouts.
	If I didn’t read the online reviews before using a fitness video, I worry about my decision.
	I often read reviews from other fitness enthusiasts to find out which fitness video make a good impression on them.
	I often consult other fitness enthusiasts’ online reviews to help me choose a good fitness video.
	To make sure I choose the right fitness video, I often read other fitness enthusiasts’ online reviews.
Information Credibility	I think fitness video information is credible.
	I think fitness video information is trustworthy.
	I think fitness video information is truthful.
	I think fitness video information is reliable.
	I think fitness video information is believable.
Perceived Usefulness	Using fitness videos helps me improve my fitness performance.
	Using fitness videos helps me reach my fitness goals faster.
	Using fitness videos helps me improve my fitness efficiency.
Exercise Intention	I intend to use this fitness video to exercise in the near future.
	I intend to use this fitness video more when I exercise.
	If I want to exercise, I will consider to use this fitness video.

## References

[1] Bull F.C., Al-Ansari S.S., Biddle S., Borodulin K., Buman M.P., Cardon G., Carty C., Chaput J.P., Chastin S., Chou R. (2020). World Health Organization 2020 guidelines on physical activity and sedentary behaviour. Br. J. Sport. Med..

[2] Durau J., Diehl S., Terlutter R. (2022). Motivate me to exercise with you: The effects of social media fitness influencers on users’ intentions to engage in physical activity and the role of user gender. Digit. Health.

[3] Jong S.T., Drummond M.J. (2020). Exploring online fitness culture and young females. Re-Thinking Leisure in a Digital Age.

[4] Vaterlaus J.M., Patten E.V., Roche C., Young J.A. (2015). # Gettinghealthy: The perceived influence of social media on young adult health behaviors. Comput. Hum. Behav..

[5] Johnston C., Davis W.E. (2019). Motivating exercise through social media: Is a picture always worth a thousand words?. Psychol. Sport Exerc..

[6] Oh Y. (2023). The Relationship between Exercise Re-Participation Intention Based on the Sports-Socialization Process: YouTube Sports Content Intervention. Behav. Sci..

[7] Kiecker P., Cowles D. (2002). Interpersonal communication and personal influence on the Internet: A framework for examining online word-of-mouth. J. Euromark..

[8] Wiedmann K.-P., Von Mettenheim W. (2020). Attractiveness, trustworthiness and expertise–social influencers’ winning formula?. J. Prod. Brand Manag..

[9] Lutkenhaus R.O., Jansz J., Bouman M.P. (2019). Tailoring in the digital era: Stimulating dialogues on health topics in collaboration with social media influencers. Digit. Health.

[10] Cai J., Zhao Y., Sun J. (2022). Factors influencing fitness app users’ behavior in China. Int. J. Hum.–Comput. Interact..

[11] Beldad A.D., Hegner S.M. (2018). Expanding the technology acceptance model with the inclusion of trust, social influence, and health valuation to determine the predictors of German users’ willingness to continue using a fitness app: A structural equation modeling approach. Int. J. Hum.–Comput. Interact..

[12] Ohanian R. (1990). Construction and validation of a scale to measure celebrity endorsers’ perceived expertise, trustworthiness, and attractiveness. J. Advert..

[13] Xiao M., Wang R., Chan-Olmsted S. (2018). Factors affecting YouTube influencer marketing credibility: A heuristic-systematic model. J. Media Bus. Stud..

[14] Reinikainen H., Munnukka J., Maity D., Luoma-Aho V. (2020). ‘You really are a great big sister’–parasocial relationships, credibility, and the moderating role of audience comments in influencer marketing. J. Mark. Manag..

[15] Santateresa-Bernat P., Sánchez-García I., Curras-Perez R. (2023). I like you, or I like what you say? Effect of influencer on tourists’ behaviours. Curr. Issues Tour..

[16] Slater M.D., Rouner D. (1996). How message evaluation and source attributes may influence credibility assessment and belief change. J. Mass Commun. Q..

[17] Duan W., Gu B., Whinston A.B. (2008). The dynamics of online word-of-mouth and product sales—An empirical investigation of the movie industry. J. Retail..

[18] Zhao J.-D., Huang J.-S., Su S. (2019). The effects of trust on consumers’ continuous purchase intentions in C2C social commerce: A trust transfer perspective. J. Retail. Consum. Serv..

[19] Davis F.D., Bagozzi R.P., Warshaw P.R. (1989). User acceptance of computer technology: A comparison of two theoretical models. Manag. Sci..

[20] Mehrabian A., Russell J.A. (1974). An Approach to Environmental Psychology.

[21] Zhang H., Lu Y., Gupta S., Zhao L. (2014). What motivates customers to participate in social commerce? The impact of technological environments and virtual customer experiences. Inf. Manag..

[22] Song Z., Liu C., Shi R. (2022). How do fresh live broadcast impact consumers’ purchase intention? Based on the SOR Theory. Sustainability.

[23] Kim A.J., Johnson K.K. (2016). Power of consumers using social media: Examining the influences of brand-related user-generated content on Facebook. Comput. Hum. Behav..

[24] Bhattacherjee A., Sanford C. (2006). Influence processes for information technology acceptance: An elaboration likelihood model. MIS Q..

[25] Hovland C.I., Janis I.L., Kelley H.H. (1953). Communication and Persuasion.

[26] Ayeh J.K. (2015). Travellers’ acceptance of consumer-generated media: An integrated model of technology acceptance and source credibility theories. Comput. Hum. Behav..

[27] Smith G. (2001). The 2001 general election: Factors influencing the brand image of political parties and their leaders. J. Mark. Manag..

[28] Djafarova E., Trofimenko O. (2019). ‘Instafamous’–credibility and self-presentation of micro-celebrities on social media. Inf. Commun. Soc..

[29] McKnight D.H., Kacmar C.J. Factors and effects of information credibility. Proceedings of the Ninth International Conference on Electronic Commerce.

[30] Erdogan B.Z. (1999). Celebrity endorsement: A literature review. J. Mark. Manag..

[31] Lê Giang Nam H.T.D. (2018). Impact of social media Influencer marketing on consumer at Ho Chi Minh City. Int. J. Soc. Sci. Humanit. Invent..

[32] Schouten A.P., Janssen L., Verspaget M. (2021). Celebrity vs. Influencer endorsements in advertising: The role of identification, credibility, and Product-Endorser fit. Leveraged Marketing Communications.

[33] Pornpitakpan C. (2004). The persuasiveness of source credibility: A critical review of five decades’ evidence. J. Appl. Soc. Psychol..

[34] Wathen C.N., Burkell J. (2002). Believe it or not: Factors influencing credibility on the Web. J. Am. Soc. Inf. Sci. Technol..

[35] Crisci R., Kassinove H. (1973). Effect of perceived expertise, strength of advice, and environmental setting on parental compliance. J. Soc. Psychol..

[36] Yadav M.S., De Valck K., Hennig-Thurau T., Hoffman D.L., Spann M. (2013). Social commerce: A contingency framework for assessing marketing potential. J. Interact. Mark..

[37] Patzer G.L. (1983). Source credibility as a function of communicator physical attractiveness. J. Bus. Res..

[38] Till B.D., Busler M. (2000). The match-up hypothesis: Physical attractiveness, expertise, and the role of fit on brand attitude, purchase intent and brand beliefs. J. Advert..

[39] Palmer C.L., Peterson R.D. (2016). Halo effects and the attractiveness premium in perceptions of political expertise. Am. Politics Res..

[40] Kamins M.A. (1990). An investigation into the “match-up” hypothesis in celebrity advertising: When beauty may be only skin deep. J. Advert..

[41] Hu H., Zhang D., Wang C. (2019). Impact of social media influencers’ endorsement on application adoption: A trust transfer perspective. Soc. Behav. Personal. Int. J..

[42] Hu X., Huang Q., Zhong X., Davison R.M., Zhao D. (2016). The influence of peer characteristics and technical features of a social shopping website on a consumer’s purchase intention. Int. J. Inf. Manag..

[43] Tien D.H., Rivas A.A.A., Liao Y.-K. (2019). Examining the influence of customer-to-customer electronic word-of-mouth on purchase intention in social networking sites. Asia Pac. Manag. Rev..

[44] Chaiken S. (1979). Communicator physical attractiveness and persuasion. J. Personal. Soc. Psychol..

[45] Carlson J., Rahman M., Voola R., De Vries N. (2018). Customer engagement behaviours in social media: Capturing innovation opportunities. J. Serv. Mark..

[46] Kim D.J., Ferrin D.L., Rao H.R. (2008). A trust-based consumer decision-making model in electronic commerce: The role of trust, perceived risk, and their antecedents. Decis. Support Syst..

[47] Vila N., Kuster I. (2011). Consumer feelings and behaviours towards well designed websites. Inf. Manag..

[48] Saima, Khan M.A. (2020). Effect of social media influencer marketing on consumers’ purchase intention and the mediating role of credibility. J. Promot. Manag..

[49] Lin J.C.-C., Lu H. (2000). Towards an understanding of the behavioural intention to use a web site. Int. J. Inf. Manag..

[50] Liao C., Palvia P., Lin H.-N. (2006). The roles of habit and web site quality in e-commerce. Int. J. Inf. Manag..

[51] Wei J., Zhang L., Yang R., Song M. (2023). A new perspective to promote sustainable low-carbon consumption: The influence of informational incentive and social influence. J. Environ. Manag..

[52] Cheung M.Y., Luo C., Sia C.L., Chen H. (2009). Credibility of electronic word-of-mouth: Informational and normative determinants of on-line consumer recommendations. Int. J. Electron. Commer..

[53] Wei P.-S., Lu H.-P. (2013). An examination of the celebrity endorsements and online customer reviews influence female consumers’ shopping behavior. Comput. Hum. Behav..

[54] Hennig-Thurau T., Walsh G., Walsh G. (2003). Electronic word-of-mouth: Motives for and consequences of reading customer articulations on the Internet. Int. J. Electron. Commer..

[55] Bickart B., Schindler R.M. (2001). Internet forums as influential sources of consumer information. J. Interact. Mark..

[56] Gu B., Park J., Konana P. (2012). Research note—The impact of external word-of-mouth sources on retailer sales of high-involvement products. Inf. Syst. Res..

[57] Huang C.-C. (2017). Cognitive factors in predicting continued use of information systems with technology adoption models. Inf. Res. Int. Electron. J..

[58] Shen X.L., Zhang K.Z., Zhao S.J. (2016). Herd behavior in consumers’ adoption of online reviews. J. Assoc. Inf. Sci. Technol..

[59] Hu Y., Shyam Sundar S. (2010). Effects of online health sources on credibility and behavioral intentions. Commun. Res..

[60] Choi K., Wang Y., Sparks B. (2019). Travel app users’ continued use intentions: It’sa matter of value and trust. J. Travel Tour. Mark..

[61] Kim D.J., Ferrin D.L., Rao H.R. (2009). Trust and satisfaction, two stepping stones for successful e-commerce relationships: A longitudinal exploration. Inf. Syst. Res..

[62] Liu C., Marchewka J.T., Lu J., Yu C.-S. (2005). Beyond concern—A privacy-trust-behavioral intention model of electronic commerce. Inf. Manag..

[63] Lee S.W., Sung H.J., Jeon H.M. (2019). Determinants of continuous intention on food delivery apps: Extending UTAUT2 with information quality. Sustainability.

[64] Alzaza N.S. (2012). Mobile Learning Services Acceptance Model among Malaysian Higher Education Students. Ph.D. Thesis.

[65] Chen S.-C., Liu M.-L., Lin C.-P. (2013). Integrating technology readiness into the expectation–confirmation model: An empirical study of mobile services. Cyberpsychol. Behav. Soc. Netw..

[66] Abubakar A.M., Ilkan M. (2016). Impact of online WOM on destination trust and intention to travel: A medical tourism perspective. J. Destin. Mark. Manag..

[67] Magno F. (2017). The influence of cultural blogs on their readers’ cultural product choices. Int. J. Inf. Manag..

[68] Lederman R., Fan H., Smith S., Chang S. (2014). Who can you trust? Credibility assessment in online health forums. Health Policy Technol..

[69] Nagy J.T. (2018). Evaluation of online video usage and learning satisfaction: An extension of the technology acceptance model. Int. Rev. Res. Open Distrib. Learn..

[70] Davis F.D. (1989). Perceived usefulness, perceived ease of use, and user acceptance of information technology. MIS Q..

[71] Hair J.F., Sarstedt M., Ringle C.M., Mena J.A. (2012). An assessment of the use of partial least squares structural equation modeling in marketing research. J. Acad. Mark. Sci..

[72] Hair J.F., Ringle C.M., Sarstedt M. (2011). PLS-SEM: Indeed a silver bullet. J. Mark. Theory Pract..

[73] Hair J.F., Hult G.T.M., Ringle C.M., Sarstedt M., Danks N.P., Ray S. (2021). Partial Least Squares Structural Equation Modeling (PLS-SEM) Using R: A Workbook.

[74] Hair Jr J.F., Sarstedt M., Hopkins L., Kuppelwieser V.G. (2014). Partial least squares structural equation modeling (PLS-SEM): An emerging tool in business research. Eur. Bus. Rev..

[75] Henseler J., Ringle C.M., Sinkovics R.R. (2009). The use of partial least squares path modeling in international marketing. New Challenges to International Marketing.

[76] Fornell C., Larcker D.F. (1981). Structural equation models with unobservable variables and measurement error: Algebra and statistics. J. Mark. Res..

[77] Bonner B.L., Baumann M.R., Lehn A.K., Pierce D.M., Wheeler E.C. (2006). Modeling collective choice: Decision-making on complex intellective tasks. Eur. J. Soc. Psychol..

[78] McGinnies E., Ward C.D. (1980). Better liked than right: Trustworthiness and expertise as factors in credibility. Personal. Soc. Psychol. Bull..

[79] Bhattacherjee A. (2001). Understanding information systems continuance: An expectation-confirmation model. MIS Q..

